# Alveolar–capillary reserve in COPD assessed by the pulmonary diffusing capacity response to an upright‐to‐supine postural change

**DOI:** 10.1113/EP093384

**Published:** 2026-05-25

**Authors:** Iben Elmerdahl Rasmussen, Stine Buus Nymand, Jacob P. Hartmann, Rie Skovly Thomsen, Helene Louise Hartmeyer, Amalie B. Andersen, Milan Mohammad, Birgitte Hanel, Jann Mortensen, Ulrik Winning Iepsen, Ronan M. G. Berg

**Affiliations:** ^1^ Centre for Physical Activity Research Copenhagen University Hospital ‐ Rigshospitalet Copenhagen Denmark; ^2^ Department of Biomedical Sciences, Faculty of Health and Medical Sciences University of Copenhagen Copenhagen Denmark; ^3^ Department of Clinical Physiology and Nuclear Medicine Copenhagen University Hospital ‐ Rigshospitalet Copenhagen Denmark; ^4^ Neurovascular Research Laboratory, Faculty of Life Sciences and Education University of South Wales Pontypridd UK; ^5^ Department of Cellular and Molecular Medicine Copenhagen University Copenhagen Denmark; ^6^ Department of Anaesthesiology and Intensive Care Copenhagen University Hospital ‐ Hvidovre Copenhagen Denmark

**Keywords:** COPD, exercise physiology, pulmonary diffusing capacity, pulmonary gas exchange

## Abstract

In patients with chronic obstructive pulmonary disease (COPD) who exhibit reduced alveolar–capillary reserve, the combined assessment of pulmonary diffusing capacity for carbon monoxide and nitric oxide (*D*_L,CO,NO_) during exercise may pose difficulties, and the transition from upright to supine posture may offer a useful alternative. A total of 50 participants (35 with COPD and 15 healthy controls) underwent measurements of *D*_L,CO,NO_ in the upright and supine postures. A subset (COPD: *n* = 12, controls: *n* = 12) also completed a 12‐week supervised high‐intensity interval training (HIIT) intervention. The reported *D*_L,CO,NO_ metrics were diffusing capacity for nitric oxide and carbon monoxide (*D*_L,NO_ and *D*_L,CO,5s_, respectively), alveolar–capillary membrane diffusing capacity (*D*_M,CO_), pulmonary capillary blood volume (*V*_C_), and alveolar volume (*V*_A_). The upright‐to‐supine change in neither *D*_L,NO_ (*P *= 0.271), nor *D*_M,CO_ (*P *= 0.068) nor *V*_A_ (*P *= 0.934) differed between groups. In contrast, the upright‐to‐supine change in *D*_L,CO5s_ was reduced in moderate and severe COPD compared with controls (control vs. moderate: median [IQR] 0.6 [0.3, 0.9] mmol/min/kPa, *P *< 0.001; control vs. severe: 0.9, [0.2, 1.5] mmol/min/kPa, *P *= 0.006), whereas it did not differ between controls and mild COPD (0.3 [−0.1, 0.7] mmol/min/kPa, *P *= 0.13). Similarly, the upright‐to‐supine *V*_C_ change was reduced in moderate and severe COPD compared with healthy controls (control vs. moderate: 8.3 [3.9, 12.8] mL, *P *< 0.001; control vs. severe: 10.7 [1.2, 20.2] mL, *P *= 0.021), but not in mild COPD (5.3 [−0.2, 10.8] mL, *P *= 0.063). The HIIT intervention had no effect on these metrics. The blunted *V*_C_ response to an upright‐to‐supine postural change in moderate‐to‐severe COPD is consistent with reduced alveolar–capillary reserve and may be useful when measurements during exercise are not possible.

## Abstract (highlights)

- **What is the central question of this study?**
  Is alveolar–capillary reserve reduced in chronic obstructive pulmonary disease (COPD) when assessed by an upright‐to‐supine postural change?
- **What is the main finding and its importance?**
  The posture‐induced increase in pulmonary capillary blood volume is specifically blunted in moderate‐to‐severe COPD, likely reflecting underlying disease‐specific pulmonary vascular alterations. Assessment of alveolar–capillary reserve through an upright‐to‐supine postural change may represent an attractive alternative to exercise‐based measurements in individuals with COPD.

## 1 INTRODUCTION

It is now well‐established that alveolar–capillary reserve, that is, the ability to increase pulmonary diffusing capacity, is reduced in chronic obstructive pulmonary disease (COPD), which is likely an important contributing mechanism to dyspnoea and reduced exercise capacity (Behnia et al., 2017; Hartmann, Nymand, Hartmeyer, Andersen, et al., 2025; Ross et al., 2020).

The reduction in alveolar–capillary reserve in COPD represents a functional correlate of the quite prominent histopathological alterations within the pulmonary microvasculature. In the healthy state, all pulmonary capillaries appear to be continually perfused with plasma (Konig et al., 1993), but only about half are perfused with red blood cells at rest (Okada et al., 1994; Wagner et al., 1999), such that the remainder can be recruited and distended for red blood cell perfusion in response to increases in cardiac output and pulmonary intravascular pressures, thus leading to an increase in diffusing capacity (Hsia, 2002). This ability is likely reduced in COPD due to alveolar–capillary rarefaction with reduced capillary length and density in conjunction with loss of alveolar–capillary surface area (Butler & Kleinerman, 1970; Wiebe & Laursen, 1998), together with remodelling of small pulmonary arteries and microvessels, characterised by intimal thickening, medial hypertrophy, smooth‐muscle proliferation, extracellular matrix deposition and perivascular inflammation (Magee et al., 1988; Peinado et al., 2008; Santos et al., 2002). Accordingly, abnormal perfusion distribution patterns may be evident from the earliest stages of disease, in some cases preceding the development of overt ventilation abnormalities (Hueper et al., 2015; Jögi et al., 2014; Mortensen & Berg, 2019). It is currently unknown whether these pulmonary vascular changes, and thus alveolar–capillary reserve, are affected by exercise training (Nymand et al., 2022), which is known to exert beneficial clinical effects in COPD (Ryrsø et al., 2018).

Experimentally, alveolar–capillary reserve can be assessed by measuring the change in pulmonary diffusing capacity in response to a standardised increase in cardiac output induced by, for example, exercise (Nymand et al., 2024; Tedjasaputra et al., 2016), infusion of an inotropic pharmacological agent (Michaelchuk et al., 2019), or a postural change with or without superimposed exercise (Madsen et al., 2023; Ross et al., 2020; Thomsen et al., 2025). In previous studies, assessments during submaximal exercise in individuals with COPD have shown the alveolar–capillary reserve to be reduced in a severity‐dependent manner (Hartmann, Nymand, Hartmeyer, Andersen, et al., 2025; Tedjasaputra et al., 2018). However, given that measuring pulmonary diffusing capacity is technically demanding and requires a sustained breath hold, this poses a particular challenge because of exertional dyspnoea during submaximal exercise in individuals with COPD (Nymand et al., 2024), critically affecting measurement quality and, consequently, its test–retest reliability, especially in individuals with moderate to severe disease (Hartmann, Nymand, Hartmeyer, Andersen, et al., 2025). Thus, in such individuals, assessment of alveolar–capillary reserve obtained at rest through postural change may represent a useful alternative. Classical integrative physiology demonstrates that transition from upright to the supine position increases central blood volume and venous return, augmenting cardiac preload and stroke volume via the Frank–Starling mechanism (Blomqvist & Stone, 1983). Furthermore, the supine position may attenuate the gravity‐dependent apical‐to‐basal perfusion gradient characteristic of the upright lung, resulting in a more homogeneous pulmonary perfusion distribution (Berg et al., 2022; Petersson et al., 2009). Experimental studies in healthy subjects confirm that seated‐to‐supine positioning increases venous return and enhances ventricular filling, reflecting central volume expansion (Pump et al., 2001a, 2001b). Consistent with these haemodynamic effects, the supine posture increases pulmonary capillary blood volume (*V*_C_) (Madsen et al., 2023). Although postural haemodynamic effects are less comprehensively characterised in COPD, available data indicate that upright‐to‐supine transition can similarly increase cardiac output and/or influence pulmonary perfusion distribution in this condition (Minh et al., 1981, 1986), and this manoeuvre has previously been applied to demonstrate reduced alveolar–capillary reserve in COPD compared with healthy individuals (Ross et al., 2020; Terzano et al., 2009).

In the present study, we compared alveolar–capillary reserve by transitioning from the upright to the supine position in individuals with COPD to that of healthy age‐ and sex‐matched controls. We hypothesised that the alveolar–capillary reserve would be reduced in COPD in a severity‐dependent manner. Furthermore, given that there is some evidence to suggest that exercise training by HIIT may affect pulmonary vascular structure and function (Nymand et al., 2022), we exploratively examined whether alveolar–capillary reserve, as determined by this protocol, was affected by a 12‐week supervised high‐intensity interval training (HIIT) programme in either group.

## 2 METHODS

### 2.1 Ethical approval

This study consists of two experimental series that were both approved by the Regional Ethical Committee of the Capital Region of Denmark (files no. H‐21021723 [Substudy 1] and H‐85658 [Substudy 2]). The two studies were performed according to the guidelines of the *Declaration of Helsinki* and were pre‐registered on ClinicalTrials.gov (NCT05583396 [Substudy 1] and NCT05552833 [Substudy 2]). Both written and oral consent were obtained from all participants prior to participation.

The study is a secondary analysis of a previously published non‐randomised controlled study, where the primary endpoint was to investigate the alveolar–capillary reserve (i.e., the change in diffusing capacity from upright rest to exercise) using the diffusing capacity for carbon monoxide and nitric oxide (*D*_L,CO,NO_) technique (Hartmann, Nymand, Hartmeyer, Andersen, et al., 2025). This secondary analysis investigated an alternative way of estimating the alveolar–capillary reserve, that is, the change from the upright to the supine position (Madsen et al., 2023).

### 2.2 Study design and setting

**FIGURE 1:**
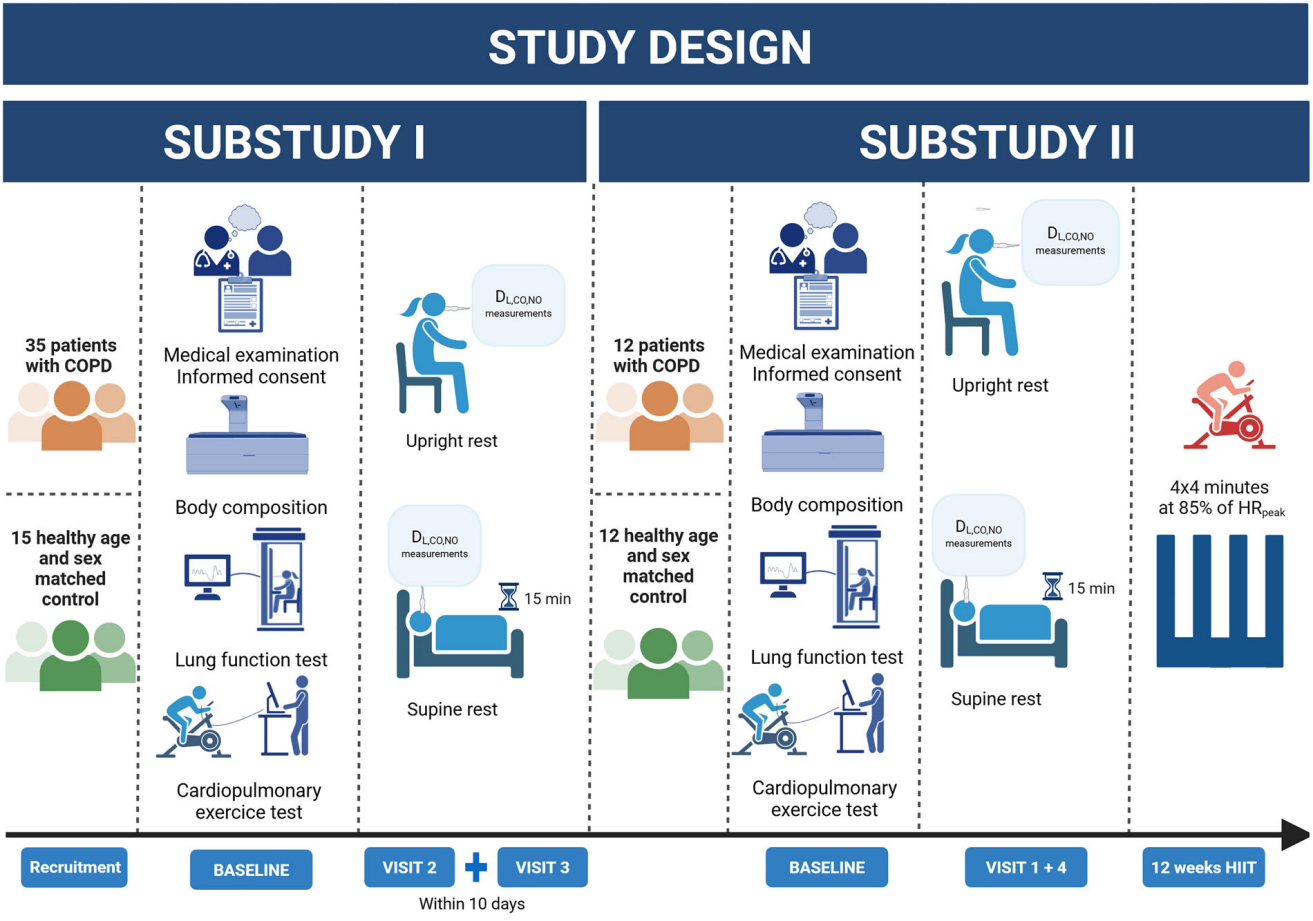
Study overview. The study comprised two sub‐studies. In both, participants underwent a baseline assessment, including medical examination, DXA scan for body composition, standardized lung function testing, and a cardiopulmonary exercise test. In Substudy 1, the participants attended two identical visits to assess the combined measurement of diffusing capacity for carbon monoxide and nitric oxide (*D*_L,CO,NO_) during upright rest and at supine rest (visit 2 and 3). A subgroup of participant from Substudy 1 also participated in Substudy 2, which consisted of a 12‐week supervised high‐intensity interval training intervention. Created in BioRender. https://BioRender.com/5tdla20.

The two experimental series are reported according to the Strengthening the Reporting of Observational Studies in Epidemiology (STROBE) Statement (von Elm et al., 2007). The study design is shown in Figure 1.

In Substudy 1, participants reported to the laboratory for three visits, including a baseline visit and two identical experimental visits. Measurements at baseline visit included a standardised lung function test, anthropometric measurements using a dual‐energy X‐ray absorptiometry (DXA) scanner, and a cardiopulmonary exercise test (CPET). On visits 2 and 3, *D*_L,CO,NO_ was measured at rest in the upright seated position followed by measurements in the supine position. Visits 2 and 3 were repeated within 10 days to calculate the reliability of the method. A subgroup from Substudy 1 performed 12 weeks of HIIT, to investigate changes in alveolar–capillary reserve. In both studies, all participants were requested to avoid consuming caffeine, alcohol, e‐cigarettes and nicotine products for at least 24 h prior to each visit Additionally, they were also instructed to abstain from strenuous physical activities for 48 h prior to the visit.

### 2.3 Participants

Participants were included from August 2022 until April 2024 at the Centre for Physical Activity Research (CFAS), Rigshospitalet, Copenhagen, Denmark with a total of 50 participants in Substudy 1, and 24 participants from Substudy 1 participated in Substudy 2. In the two studies, 35 patients diagnosed with mild to severe COPD classified as stage I to III by the Global Initiative for Chronic Obstructive Lung Disease (GOLD) guidelines were included (Agustí et al., 2023) together with 15 healthy age‐ and sex‐matched controls. The inclusion criteria for COPD patients were: (1) both sexes, (2) age between 45 and 80 years, (3) a forced expiratory volume in 1 s (FEV_1_) to forced vital capacity (FVC) ratio below 0.7, (4) a Modified Medical Research Council (mMRC) score between 0 and 3, and (5) a resting arterial oxygen saturation above 90%. For the healthy age‐ and sex‐matched participants, the criteria were: (1) both sexes, (2) age between 45 and 80 years, (3) a normal FEV_1_ and FVC and FEV_1_/FVC ratio above 0.7, and (4) a normal pulmonary diffusing capacity for carbon monoxide corrected for haemoglobin and based on a 10‐s breath‐hold (*D*_L,CO,10s_) corrected for haemoglobin. Exclusion criteria which applied to all participants included heart failure, ischaemic heart disease or arrhythmias, peripheral arterial disease, renal or hepatic impairment, cancer, current pregnancy, or recent symptoms of any illness within the past 2 weeks. In Substudy 2, additional exclusion criteria were active smoking and not fulfilling the physical activity recommendations by The Danish Health Authority for both groups. In Substudy 2, healthy controls were individually matched based on sex and age (±3 years) to the patients with COPD.

### 2.4 Measurements

A medical examination (including blood pressure measurements and ECG) and a standardised lung function test were conducted prior to inclusion. If inclusion criteria were fulfilled, a whole‐body DXA scan and maximal incremental CPET were performed to obtain body composition and peak oxygen uptake (${\dot{V}}_{O_{2}\mathrm{peak}}$). In Substudy 2, participants performed the measurements before and after completing the HIIT intervention. In both studies, FEV_1_, FVC, FEV_1_/FVC ratio, total lung capacity (TLC), residual volume (RV) and *D*_L,COc,10s_ were reported as absolute values and as a percentage of predicted according to standard reference equations (Stanojevic et al., 2022) and were obtained by lung function testing as described in detail elsewhere (Hartmann, Nymand, Hartmeyer, Andersen, et al., 2025). Likewise, an incremental CPET until intolerance was performed as described elsewhere (Hartmann, Nymand, Hartmeyer, Andersen, et al., 2025), that is, on a cycle ergometer (E839, Monark Exercise AB, Vansbro, Sweden). Ten minutes after the CPET, a verification bout was performed at 110% of maximal workload until intolerance. A true maximal oxygen uptake (${\dot{V}}_{O_{2}\max}$) was accepted if the ${\dot{V}}_{O_{2}}$ obtained during the CPET did not exceed the ${\dot{V}}_{O_{2}}$ obtained in the verification bout (VB) by >3.5%, corresponding to the in‐house coefficient of variance (CV), which is similar to that reported by others (Schneider et al., 2019). If a true ${\dot{V}}_{O_{2}\max}$ was not confirmed, ${\dot{V}}_{O_{2}\mathrm{peak}}$ was reported as the highest obtained ${\dot{V}}_{O_{2}}$_._

#### 2.4.1 Dual test gas diffusing capacity measurements

*D*_L,CO,NO_ was determined by the single‐breath manoeuvre (Jaeger MasterScreen PFT pro, CareFusion, Höchberg, Germany) in accordance with established guidelines. Manoeuvres were first performed in the upright resting position followed by 15 min relaxation in a supine position. After the 15 min, the manoeuvres were performed in the supine position. The participants performed four tidal breaths and then exhaled to RV followed by a maximal inhalation of a test gas mixture (0.28% CO, 9.3% He, 20.9% O_2_ and 69.52% N_2_ mixed with 800 ppm NO/N_2_). The participants were then instructed to do a 5‐s breath‐hold, and then exhale to RV, from where the gas samples were collected and analysed. Each *D*_L,CO,NO_ manoeuvre was separated by at least 4 min and assessed for acceptability and repeatability (Nymand et al., 2024). The manoeuvre was accepted if the inspired volume was >90% of the FVC or vital capacity (VC) measured on the same visit or a VC >85% of the FVC or VC together with alveolar volume (*V*_A_) within 200 mL or 5% of the largest *V*_A_ obtained from another acceptable manoeuvre (Zavorsky et al., 2017). In addition, the breath‐hold had to be stable for 4–8 s with no evidence of leaks or the presence of Valsalva or Müller manoeuvres. The repeatability criteria required two acceptable manoeuvres with values within <5.8 mmol min^−^^1^ kPa^−^^1^ for diffusing capacity for nitric oxide (*D*_L,NO_) and <1 mmol min^−^^1^ kPa^−^^1^ for diffusing capacity for carbon monoxide with 5‐s breath‐hold (*D*_L,CO,5s_). A maximum of three manoeuvres were performed in each position. The *D*_L,CO,NO_ technique permits up to 12 manoeuvres in the same session without affecting *D*_L,CO,5s_ (Zavorsky et al., 2017), and up to 22 manoeuvres without affecting *D*_L,NO_ (Zavorsky, 2013). Each manoeuvre was specifically evaluated to the experimental setting and reported as based on these criteria as reported in detail elsewhere (Hartmann, Nymand, Hartmeyer, Andersen, et al., 2025; Nymand et al., 2024).

#### 2.4.2 HIIT intervention

As described in detail elsewhere (Hartmann, Nymand, Hartmeyer, Andersen, et al., 2025), a subgroup of 12 COPD patients and 12 control participants completed a 12‐week intervention with three supervised 4 × 4 HIIT sessions per week performed on a cycle ergometer, a training protocol that has previously been shown to be feasible in people with COPD (Helgerud et al., 2010; Nymand et al., 2023).

### 2.5 Calculations

The predicted ${\dot{V}}_{O_{2}\mathrm{peak}}$ related to sex was calculated using a standard formula (Myers et al., 2017).

The alveolar–capillary membrane diffusing capacity for carbon monoxide (*D*_M,CO_) and *V*_C_ were calculated from each *D*_L,CO,NO_ manoeuvre, as based on the Roughton–Forster equation. This was based on empirically derived constants as recommended by the ERS task force: the diffusivity ratio α = $\frac{D_{M,\mathrm{NO}}}{D_{M,\mathrm{CO}}}$ was assumed to be 1.97 and *k* = $\frac{\theta_{\mathrm{NO}}}{\theta_{\mathrm{CO}}}$, where where θ is the specific blood conductance for the given gas, that is, 1.51 mmol mL min^−1^ kPa^−1^ for θ_NO_, and with θ_CO_ being calculated as described elsewhere (Munkholm et al., 2018). On this bases, the following was calculated:

$$\;D_{M,\mathrm{CO}}=\frac{\frac{1}{\alpha }\;-\;\frac{1}{k}}{\frac{1}{D_{L,\mathrm{NO}}\;}-\;\frac{1}{k\;\cdot D_{L,\mathrm{CO},5s}}}\;V_{C}=\frac{\left(\frac{1}{\theta_{\mathrm{CO}}}\right)\left(1-\frac{\alpha }{k}\right)}{\frac{1}{D_{L,\mathrm{CO},5s\;}}\;-\;\frac{\alpha }{D_{L,\mathrm{NO}}}}$$

Full methodological details have been published elsewhere (Hartmann, Nymand, Hartmeyer, Andersen, et al., 2025).

#### 2.5.1 Sample sizes

In Substudy 1, CV of *D*_L,NO_ during supine manoeuvres in healthy young individuals has previously been determined to be 2.2% (Madsen et al., 2023). Assuming a similar CV for patients with COPD, *n* ≥ 9 was assumed to be required to show a 5% difference in CV for *D*_L,NO_ between groups with a statistical power of 90%. However, CV is most likely higher in people of higher age especially in those with COPD. Inclusion of up to 45 COPD patients, with an expected prevalence of at least 10 for each severity stage, was planned during the study period. Substudy 2 should be considered a pilot study, with consensus established on a sample size of 12 vs. 12 (Julious, 2005).

### 2.6 Statistics

The statistical analyses were conducted using R statistical software (version 4.3.3; R Foundation for Statistical Computing, Vienna, Austria). Histograms and quantile–quantile (QQ) plots were visually inspected to check for normality of the data. Baseline characteristics are reported as means (standard deviation (SD)) or medians [25th percentile, 75th percentile].

In Substudy 1, data were analysed using linear mixed effects regression (lme4 package, version 1.1‐35.3) with fixed effects for variables such as condition (upright, supine), severity (control, mild‐COPD, moderate‐COPD, severe‐COPD), group (control, COPD), sex, age and height, and random intercepts for participant ID. The results are presented as estimated marginal means or mean differences with 95% confidence intervals (emmeans package, version 1.10.1). For each participant, upright‐to‐supine changes in *D*_L,NO_, *D*_L,CO,5s_, *D*_M,CO_, *V*_C_ and *V*_A_ were averaged across day 1 and day 2, and Pearson correlations with ${\dot{V}}_{O_{2}\mathrm{peak}}$ were calculated separately for COPD patients and the control group.

In Substudy 2, the same statistical model was employed; however, only age and height were included as covariates. Outcomes are reported as estimated marginal means or mean differences with associated 95% confidence intervals. Interaction terms were incorporated when appropriate, and assumptions of the models were evaluated visually using plots of fitted values versus residuals. When necessary to meet these assumptions, outcome variables underwent log transformation. In such instances, estimated marginal means were back‐transformed to the original scale, and mean differences were expressed as percentage changes, derived from the ratio of geometric means or from ratios of ratios. No adjustments for multiplicity were applied to *P*‐values or 95% confidence intervals, and statistical significance was defined as α < 0.05 (two‐sided).

Absolute and relative reliability (Hartmann et al., 2023) were examined using the publicly available *calcrel* function from the *clintools* package, version 0.9.8 (Olsen et al., 2022). Absolute reliability was quantified using the smallest real difference (SRD), which reflects the maximum expected difference between two measurements in 95% of cases, estimated via a one‐way analysis of variance (Vaz et al., 2013). The primary metric of relative reliability was the CV, obtained from the distribution of estimated means, standard deviations and residual variances in a linear mixed model, with 95% confidence intervals (Liu, 2012). As a secondary measure, the two‐way mixed‐effects single‐measurement absolute‐agreement intraclass correlation coefficient (ICC) was also reported.

All test–retest analyses were conducted exclusively on complete‐case datasets. Comparisons between reliability metrics were performed via bootstrapping using the *comparerel* function in the *clintools* package. Across 1000 bootstrap iterations, the differences between measures were computed along with corresponding confidence intervals and *P*‐values.

## 3 RESULTS

### 3.1 Participant characteristics

The baseline characteristics separated by groups of COPD severity and control group are shown in Table 1. The demographics of the participants were similar, but the COPD group had a lower FEV_1_, FVC, FEV_1_/FVC ratio and *D*_L,COc,10s_ compared to the control group. Furthermore, the cardiorespiratory fitness was lower in the COPD group, indicated by a lower ${\dot{V}}_{O_{2}\mathrm{peak}}$ and peak workload (*W*_Lpeak_) as reported elsewhere (Hartmann, Nymand, Hartmeyer, Andersen, et al., 2025). The quality criteria for all reported *D*_L,CO,NO_ assessments are provided in Appendix Table A1.

**TABLE 1:** Baseline characteristics.

	Healthy controls	GOLD I	GOLD II	GOLD III	ANOVA *P*
*n*	15	10	20	5	
Sex (f/m)	7/8	4/6	13/7	2/3	
Age (years)	65 (5)	64 (7)	68 (6)	72 [54, 73]	0.250
Weight (kg)	79 (12)	81 (17)	76 (16)	84 [67, 90]	0.984
Height (cm)	176 (9)	174 (12)	168 (13)	170 [150,174]	0.165
BMI (kg/m^2^)	25 (3)	27 (4)	27 (4)	29 [27, 30]	0.218
FEV_1_ (L)	3.7 (1)	2.7 (1)^*^	1.7 (1)^* #^	1.2 [0.9, 1.4]^* #^	<0.0001
FEV_1_ (% of predicted)	119 (13)	86 (6)^*^	63 (9)^*^	45 [33, 49]^*, #^	<0.0001
FVC (L)	4.8 (1)	4.7 (1)	3.3 (1)^*^	3.3 [1.6, 3.6]^*^	0.004
FVC (% of predicted)	120 (12)	114 (15)^*^	96 (15)^* #^	91 [58, 100]^*# §^	<0.0001
FEV_1_/FVC	0.75 (0.1)	0.59 (0.1)	0.51 (0.1)^*#^	0.39 [0.3, 0.6]^*, #^	<0.0001
TLC (L)	7.0 (1)	7.4 (2)	6.2 (1)	7.0 [5.2, 8.2]	0.139
TLC (% of predicted)	109 (10)	116 (13)	107 (16)	130 [123, 130]	0.303
RV (L)	2.4 (0.4)	2.8 (0.7)	2.9 (0.8)	3.5 [3.3, 4.8]^*^	0.002
RV (% of predicted)	101 (14)	124 (19)	132 (36)	179 [176, 184]^*^	<0.0001
*D*_LCOc,10s_ (mmol min^−1^ kPa^−1^)	8.7 (2)	7.3 (2)	5.5 (2)^*^	5.3 [4.2, 5.8]^*^	<0.0001
*D*_LCOc,10s_ (% of predicted)	95.3 (12)	84.8 (16)	71.1 (16)^*^	59.8 (11)*	<0.0001
${\dot{V}}_{O_{2}\mathrm{peak}}$ (ml/min/kg)	29.2 (6.1)	25.3 (4.6)	21.0 (5.4)^*^	17.4 [15.0, 19.0]^*^	<0.0001

*Note*: Data are presented as means (standard deviation) or medians [interquartile range]. A one‐way ANOVA was used to test differences between groups. In cases with a significant difference, a *post hoc* Tukey with a Holm–Bonferroni adjustment analysis was performed to test between group differences with ^*^ *P *< 0.05 compared to Healthy Control, ^#^ *P *< 0.05 compared to Gold I, ^§^ *P *< 0.05 compared to Gold II. Abbreviations: GOLD, Global Initiative for Chronic Obstructive Lung Disease; FEV_1_, forced expiratory volume in 1 s; FVC, forced vital capacity; *D*_L,COc,10s_, pulmonary diffusing capacity for carbon monoxide corrected for haemoglobin with a 10‐s breath‐hold; RV, residual volume; TLC, total lung capacity; ${\dot{V}}_{O_{2}\mathrm{peak}}$, peak oxygen uptake.

### 3.2 Alveolar–capillary reserve

Only patients with valid measurements on both study visits were included in the analysis (COPD: *n* = 33 upright, *n* = 31 supine; controls: *n* = 15 for both positions). Absolute values on all *D*_L,CO,NO_ metrics, as well as data on between‐day internal consistency and absolute and relative test–retest reliability indices are provided in Appendix Tables A2 and A3, respectively.

**FIGURE 2:**
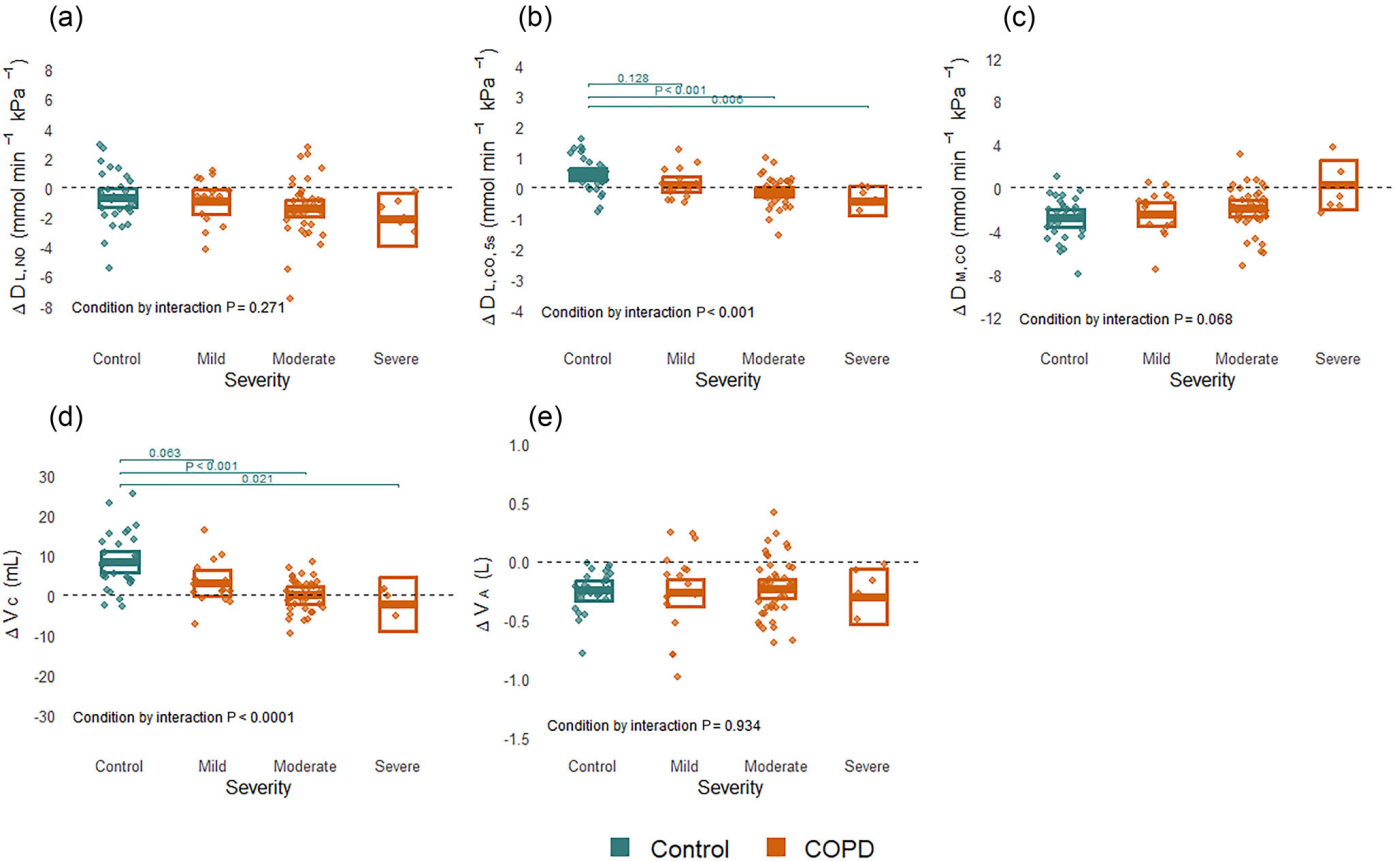
Alveolar–capillary reserve measured by an upright‐to‐supine postural change. Upright‐to‐supine changes in *D*_L,CO,NO_ metrics. Each dot represents a measurement, and the plot includes measurements conducted on both study visits. *D*_L,CO,NO_ estimates for healthy controls (green, *n* = 15) and COPD (orange, mild COPD: *n* = 2 × 9, moderate COPD: *n* = 2 × 20 severe COPD: *n* = 2 × 4). The boxes show the estimated mean differences (95% confidence interval) when controlled for sex, age and height. The reported *P*‐values are the condition by severity interaction; if below 0.05, the *P*‐values reported are for the comparisons between a given COPD severity and controls. COPD, chronic obstructive pulmonary disease; *D*_L,NO_, diffusing capacity for nitric oxide; *D*_L,CO,5s_, diffusing capacity for carbon monoxide with 5‐sec breath‐hold; *D*_M,CO_, alveolar–capillary membrane diffusing capacity for carbon monoxide; *V*_A_, alveolar volume; *V*_C_, pulmonary capillary blood volume.

The upright‐to‐supine changes in all *D*_L,CO,NO_ metrics are shown in Figure 2. The postural change from upright to supine decreased *D*_L,NO_ similarly in healthy controls and across all COPD severity groups (Figure 2a), whereas *D*_L,CO,5s_ increased only in healthy controls and those with mild COPD (Figure 2b). Overall, the latter response was less pronounced in COPD, with no significant differences between COPD severities. For *D*_M,CO_, the condition‐by‐interaction effect did not reach statistical significance at the 0.05 level (*P *= 0.078) (Figure 2c). For *V*_C_, an increase was observed only in healthy controls and mild COPD, being overall less pronounced in COPD, but with no significant differences between COPD severities (Figure 2d). *V*_A_ decreased from upright‐to‐supine in all groups, with no difference between groups (*P *= 0.934).

Apart from *V*_A_, none of the upright‐to‐supine changes in any of the *D*_L,CO,NO_ metrics correlated significantly with ${\dot{V}}_{O_{2}\mathrm{peak}}$ (*D*_L,NO_: COPD, *r* = −0.179, *P *= 0.326; controls, *r* = 0.147, *P *= 0.601; *D*_L,CO,5s_: COPD, *r* = −0.089, *P *= 0.628; controls, *r* = 0.191, *P *= 0.496; *D*_M,CO_: COPD, *r* = −0.202, *P *= 0.267; controls, *r* = −0.116, *P *= 0.681; *V*_C_: COPD, *r* = −0.012, *P *= 0.949; controls, *r* = 0.089, *P *= 0.752; *V*_A_: COPD, *r* = −0.381, *P* = 0.032 controls, *r* = −0.042, *P *= 0.882).

### 3.3 Effect of HIIT on alveolar–capillary reserve

**FIGURE 3:**
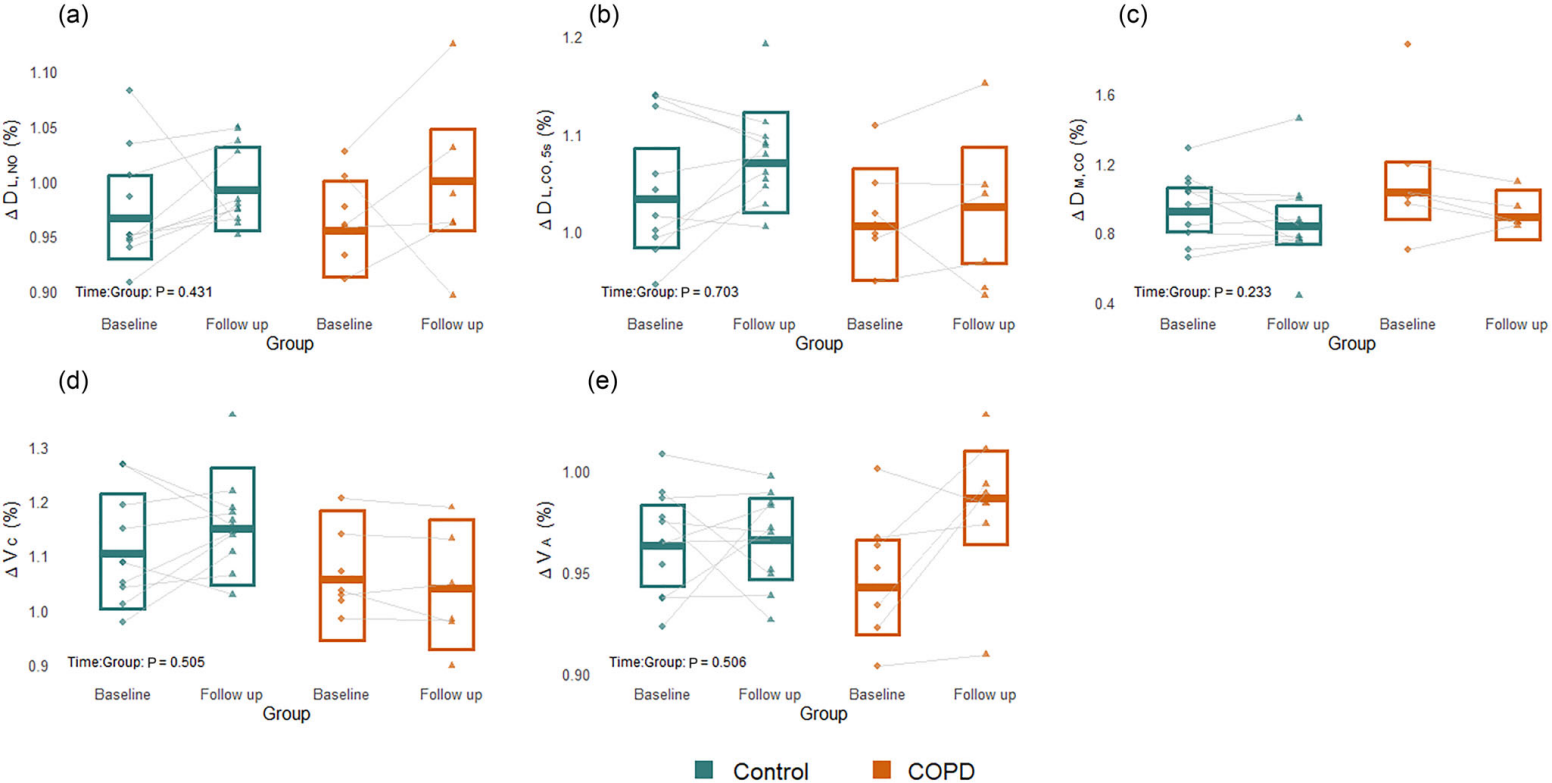
Alveolar–capillary reserve before and after a 12‐week HIIT intervention. Upright‐to‐supine changes in *D*_L, CO, NO_ metrics before and after a 12‐week HIIT intervention. COPD (orange, *n* = 11) and control group (green, *n* = 12) at baseline (circle) and at follow‐up (triangle). Each dot represents a measurement. The boxes show the estimated mean (95% confidence interval) averaged over sex. The grey lines connect the baseline and follow‐up measurement for each participant. COPD, chronic obstructive pulmonary disease; *D*_L, CO, 5s_, diffusing capacity for carbon monoxide with 5‐sec breath‐hold; *D*_M, CO_, alveolar–capillary membrane diffusing capacity for carbon monoxide; *D*_L,NO_, diffusing capacity for nitric oxide; HIIT, high intensity interval training; *V*_A_, alveolar volume; *V*_C_, pulmonary capillary blood volume.

The individuals who completed the HIIT intervention did so with an average adherence of 95 ± 6% in the healthy controls and 96 ± 4% in COPD. ${\dot{V}}_{O_{2}\mathrm{peak}}$ increased in both healthy controls (256 [120, 393] mL/min, *P* = 0.006) and COPD (192 [55, 329] mL/min, *P *= 0.008), with a greater increase in the former group (time–group interaction, *P *= 0.037). None of the upright‐to‐supine changes in *D*_L,CO,NO_ metrics were affected by the training intervention in either COPD or healthy controls (Figure 3). The estimated marginal mean difference between baseline and follow‐up in COPD vs. controls was 0.7 [−1.3, 2.6] vs. : 1.0 [−0.7, 2.7] mmol min^−^^1^ kPa^−^^1^ for *D*_L,NO_, 0.0 [−0.1, −0.1] vs. 0.0 [0.0, −0.1] mmol min^−^^1^ kPa^−^^1^ for *D*_L,CO,5s_, −0.1 [−0.4, 0.1] vs. −0.1 [−0.3; 0.1] mmol min^−^^1^ kPa^−^^1^ for *D*_M,CO_, −0.0 [−0.2, 0.1] vs. 0.0 [−0.1, 0.2] mL for *V*_C_, and 0.2 [0.0, 0.4] vs. 0.03 [−0.1, 0.2] mL for *V*_A_.

## 4 DISCUSSION

Overall, this study showed that alveolar–capillary reserve assessed by an upright‐to‐supine postural change exhibits a blunted *V*_C_ response in COPD that is unaffected by a 12‐week HIIT intervention.

It is worth noting that the change in *D*_L,CO,NO_ metrics observed when moving from the upright to the supine position in healthy controls differs from previously reported findings in healthy young individuals, in which the supine position increases both *D*_M,CO_ and *V*_C_ (Madsen et al., 2023), whereas in the ∼30 years older healthy controls examined here, *D*_M,CO_ decreases while *V*_C_ increases. In both cases, the postural change triggers a redistribution of blood from below the venous indifference point, particularly from the splanchnic vascular bed and lower extremities to the pulmonary vasculature. However, whereas the increase in *D*_M,CO_ observed in healthy young individuals indicates concomitant pulmonary capillary recruitment of previously unperfused capillaries, the decrease in *D*_M,CO_ observed here probably reflects the compression of the dorso‐caudal lung regions, caused by cranial displacement of the abdominal contents in the supine position (Berg et al., 2022). This appears to be more pronounced in elderly individuals, thereby leading to a reduction in the alveolar–capillary membrane area available for gas exchange. Thus, the concomitant increase in *V*_C_ in both healthy controls and patients with mild COPD in the present study suggests that while fewer capillaries are perfused in the supine position, the remaining perfused capillaries undergo distension, from an elliptical to a more circular configuration. Similar changes have previously been reported in both mild COPD and healthy elderly controls (Ross et al., 2020).

A novel finding in the present study is that the increase in *V*_C_ in response to an upright‐to‐supine postural change appears specifically blunted in moderate‐to‐severe COPD. This aligns with a previous study showing that the pulmonary blood volume response to pharmacologically induced hyperaemia via adenosine infusion is reduced in individuals with COPD compared with healthy controls, although it was not possible to assess the dependence of this response on COPD severity in this study (Hartmann et al., 2024). The resultant state of so‐called ‘central hypovolaemia’ may contribute not only to impaired gas exchange in COPD (Tedjasaputra et al., 2018), but also to autonomic dysregulation of the cardiovascular system, a recognised complication of COPD (Andreas et al., 2005; Haarmann et al., 2016; Heindl et al., 2001). Mechanistically, when interpreted in the context of the absence of a concomitant *D*_M,CO_ increase, these findings suggest that the capacity for pulmonary capillary distension and/or venous return is reduced. Although it is well established that the compliance of the pulmonary arterial system is reduced from the early stages of COPD (Agoston‐Coldea et al., 2018; Ertan et al., 2013; Mills et al., 2008), it is likely that this also applies to the microvasculature, though the extent remains unknown. In terms of blood volume, classical studies found no overt changes in COPD (Cocking & Darke, 1972); however, to our knowledge, this has not been investigated in COPD patients with contemporary risk factor profiles and treatments. The lack of between‐group statistical significance was somewhat unexpected, given that the primary analysis of this study revealed a COPD severity‐dependent reduction in alveolar–capillary reserve, assessed as the *D*_L,NO_ response to submaximal exercise (Hartmann, Nymand, Hartmeyer, Andersen, et al., 2025). The absence of a severity‐dependent difference may thus indicate that the *V*_C_ increase in response to the supine posture is a less sensitive measure of alveolar–capillary reserve or pulmonary vascular function as such. Accordingly, a previous study likewise found no difference in the upright‐to‐supine change in *V*_C_ between individuals with mild COPD and healthy controls; however, when exercise was superimposed on the supine position, the *V*_C_ increase was blunted in the mild COPD group (Ross et al., 2020). Additional measurements with superimposed exercise in the supine position could therefore possibly have revealed more subtle, severity‐dependent between‐group differences in the present study.

**FIGURE A1:**
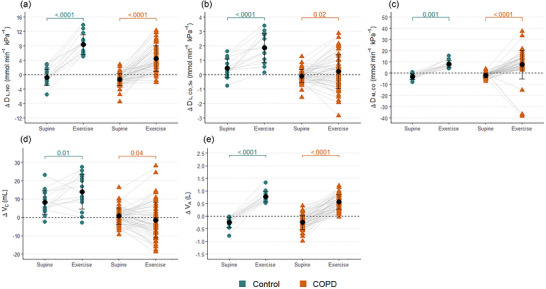
Alveolar–capillary reserve assessed by upright‐to‐supine vs. rest‐to‐submaximal exercise protocols. Changes from upright‐to‐supine and rest‐to‐exercise assessed with the *D*_L,CO,NO_ technique in chronic obstructive pulmonary disease (COPD), orange, *n* = 65) and healthy controls (green, *n* = 30). Each dot represents a measurement from a participant, and both visits was included in the analysis. The black dot and line show the mean ± SD and grey lines connect measurements from the same participant across conditions. Differences between conditions for each group were assessed using a linear mixed‐effects model with condition, group, and their interaction as fixed effects and participant as a random intercept; pairwise comparisons were performed using estimated marginal means. COPD, chronic obstructive pulmonary disease; *D*_L,CO,5s_, diffusing capacity for carbon monoxide with 5‐sec breath‐hold; *D*_L,NO_, diffusing capacity for nitric oxide; *D*_M,CO_, alveolar–capillary membrane diffusing capacity for carbon monoxide; *V*_A_, alveolar volume; *V*_C_, pulmonary capillary blood volume.

**FIGURE A2:**
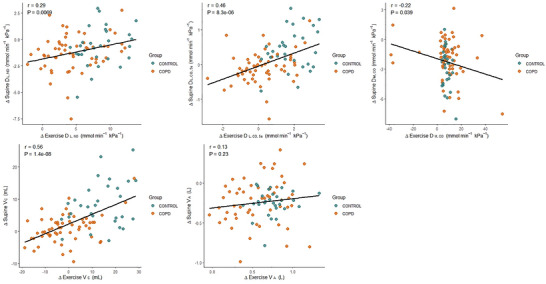
Correlations between alveolar–capillary reserve metrics assessed by upright‐to‐supine vs. rest‐to‐submaximal exercise protocols. Changes from upright‐to‐supine and rest‐to‐exercise assessed with the *D*_L,CO,NO_ technique in chronic obstructive pulmonary disease (COPD; orange, *n* = 65) and healthy controls (green, *n* = 30). Each dot represents a measurement from a participant, and both visits was included in the analysis. The corresponding Pearson's correlation coefficient between the upright‐to‐supine and rest‐to‐exercise changes are provided. COPD, chronic obstructive pulmonary disease; *D*_L,CO,5s_, diffusing capacity for carbon monoxide with 5‐sec breath‐hold; *D*_L,NO_, diffusing capacity for nitric oxide; *D*_M,CO_, alveolar–capillary membrane diffusing capacity for carbon monoxide; *V*_A_, alveolar volume; *V*_C_, pulmonary capillary blood volume.

*D*_L,CO,NO_ measurements obtained during submaximal exercise, which are often technically challenging, have previously been reported to lead to invalid results in some cases of moderate and severe COPD (Hartmann, Nymand, Hartmeyer, Andersen, et al., 2025). Under such conditions, test–retest reliability may furthermore be critically affected (Hartmann, Nymand, Hartmeyer, Andersen, et al., 2025), and the assessment of alveolar–capillary reserve at rest through postural change may thus represent an attractive alternative. However, while a postural change to the supine position may provide a standardised alternative approach to investigating the underlying phenomenon, that is, the functional impact of disease‐specific pulmonary vascular changes on the ability to recruit and distend pulmonary capillaries, the responses of the different *D*_L,CO,NO_ metrics differ between these two approaches, as they will differ between exercise intensities, owing to differing effects on cardiac output, *V*_A_, and perhaps on the pulmonary perfusion distribution. In Appendix Figure A1, the upright‐to‐supine changes in all *D*_L,CO,NO_ metrics are compared with those obtained at rest and during exercise in the same individuals. The changes in all metrics are less pronounced in response to the supine posture than during submaximal exercise; however, the pattern of a blunted *V*_C_ response in COPD is evident in both protocols. Accordingly, and as shown in Appendix Figure A2, the changes in all metrics apart from *V*_A_ exhibited statistically significant correlations between the upright‐to‐supine and rest‐to‐submaximal exercise protocols with *V*_C_ exhibiting the highest Pearson's correlation coefficient (*r* = 0.56).

In terms of absolute and relative reliability, assessed by SRD and CV, these were similar or even slightly better in the supine than in the upright position, and largely comparable between groups (see Appendix Table A3). Indeed, measurements obtained in individuals with COPD in the supine position demonstrated CV estimates similar to that for young healthy individuals in the same posture (Madsen et al., 2023). Compared to submaximal exercise in the same COPD patients, these CV estimates were comparable for *D*_L,NO_ (4.5% vs. 5.9%) and *V*_C_ (9.7% vs. 10.7%), and lower for *D*_L,CO,5s_ (5.4% vs. 8.0%) and *D*_M,CO_ (6.9% vs. 29.7%), respectively (Hartmann, Nymand, Hartmeyer, Andersen, et al., 2025). With these reliability estimates in mind, and despite a potentially lower sensitivity as a measure of alveolar–capillary reserve, the aforementioned finding that a reduced *V*_C_ response was specifically observed in moderate‐to‐severe COPD supports the use of this protocol as an attractive alternative when measurements during submaximal exercise are not feasible.

The HIIT intervention in the present study had no effect on the upright‐to‐supine changes in any of the *D*_L,CO,NO_ metrics in either group. Accordingly, the results from the primary analysis of this study assessing the alveolar–capillary reserve assessed during submaximal exercise is reported elsewhere (Hartmann, Nymand, Hartmeyer, Andersen, et al., 2025). Thus, changes in alveolar–capillary reserve were clearly not the primary driver of the observed marked increase in ${\dot{V}}_{O_{2}\mathrm{peak}}$ in either group. Accordingly, while HIIT probably primarily exerts this effect inducing structural and functional cardiac adaptations, it rather seems to increase the blood flow response to exercising muscle in COPD, according to previously published findings from this study (Hartmann, Caldwell, et al., 2026, Hartmann, Nymand, Hartmeyer, Ryrsø, et al., 2025). Another contributing factor may be intrinsic skeletal muscle adaptations, as previous studies have demonstrated that HIIT induces increases in mitochondrial oxidative capacity, capillarization and/or muscle fibre cross‐sectional area in both healthy individuals and patients with COPD following similar training interventions (Dohlmann et al., 2018; Vogiatzis et al., 2005). In any event, randomised trials are warranted to clarify whether the changes in alveolar–capillary reserve observed in COPD reflect an irreversible limitation or a functionally reversible component responsive to exercise training.

There are several limitations. Participants were matched on sex and age; however, matching based on cardiorespiratory fitness would possibly have been more appropriate. This is relevant given the uncertainty as to whether changes in alveolar–capillary reserve are primarily a consequence of the disease itself or of deconditioning. Matching ${\dot{V}}_{O_{2}\mathrm{peak}}$ poses a challenge, as healthy individuals typically demonstrate higher ${\dot{V}}_{O_{2}\mathrm{peak}}$, a difference also evident in this study. Pulmonary hypertension was not evaluated in the present study, which may represent a potential confounding factor in the observed upright‐to‐supine responses within this population. No statistically significant differences in *V*_C_ were detected across the COPD severity groups; however, the analysis of disease severity was likely underpowered, as only five participants with severe COPD were included. This limited sample size may have increased the risk of a type II error (Berg et al., 2024).

Methodological limitations related to the *D*_L,CO,NO_ technique used here must also be considered. Systematic errors in *D*_M,CO_ and *V*_C_ measurements may arise from the underlying assumptions and empirically derived constants (Borland & Hughes, 2020). For example, the commonly adopted diffusivity ratio α of 1.97, used here to denote the ratio of the physical solubilities of NO and CO in tissue, has been questioned. Some authors have proposed higher α values to reconcile inconsistencies between different measurement techniques; however, these proposals are largely discounted, as they imply departures from the established physical diffusivity ratio associated with θ_NO_, leading to internally inconsistent α estimates (Zavorsky et al., 2017). In addition, θ_NO_ is assumed to be finite, despite its historical treatment as infinite owing to the rapid reaction of NO with free haemoglobin. More recent discussion and experimental work have revised this assumption, demonstrating that θ_NO_ is indeed finite (Borland & Hughes, 2020). The value adopted in the present study represents the best available estimate and shows good agreement with theoretical considerations as well as extensive in vitro and in vivo data (Zavorsky et al., 2017). Furthermore, the underlying equations describing θ_CO_ are based on empirical constants obtained at a pH of 7.4, superseding earlier values derived under less accurate and non‐physiological pH conditions (Forster, 1987). For physiological interpretation, it should nevertheless be recognised that θ_CO_ and oxygen tension are treated as constant, even though alveolar oxygen tension varies with changes in $F_{IO_{2}}$, ambient pressure and alveolar ventilation. Although such variability may occur, the relationships underpinning these empirical constants and derivations are not modified by changes in oxygen tension; moreover, attempts to adjust for oxygen tension empirically have previously been shown to markedly reduce test–retest reliability (Madsen et al., 2023). Accordingly, no such adjustments are applied here. Pending more conclusive evidence, current recommendations support retaining the established empirical constants and derivations for α, θ_NO_ and θ_CO_.

In conclusion, by using the *D*_LCO,NO_ technique, the *V*_C_ response to an upright‐to‐supine transition was found to be attenuated in moderate‐to‐severe COPD, potentially reflecting underlying pulmonary vascular dysfunction with concomitantly reduced alveolar–capillary reserve. While a postural change is likely less sensitive for detecting COPD severity‐dependent changes in alveolar–capillary reserve than submaximal exercise, it may represent an attractive alternative, offering similar or even better test–retest reliability when exercise measurements are not feasible. Although the *V*_C_ response to an upright‐to‐supine transition was unaffected by a 12‐week HIIT intervention, further studies are required to determine whether the pulmonary vascular dysfunction in COPD is entirely irreversible and unresponsive to exercise training or other pulmonary rehabilitation interventions.

## AUTHOR CONTRIBUTIONS

Iben Elmerdahl Rasmussen: Data collection, data analysis, data interpretation, first draft, revisions. Stine Buus Nymand: Design, data collection, data analysis, data interpretation, figures, first draft, revisions. Jacob P. Hartmann: Design, data collection, data analysis, data interpretation, revisions. Helene Louise Hartmeyer: Data collection, data interpretation, revisions. Amalie B. Andersen: Data collection, data interpretation, revisions. Milan Mohammad: Data collection, data analysis, revisions. Rie Skovly Thomsen: Data collection, data analysis, data interpretation, revisions. Birgitte Hanel: data interpretation, revision. Jann Mortensen: data interpretation, revision. Ulrik Winning Iepsen: Design, revisions. Ronan M. G. Berg: Conception, design, data interpretation, first draft, revisions, supervision. All authors have read and approved the final version of this manuscript and agree to be accountable for all aspects of the work in ensuring that questions related to the accuracy or integrity of any part of the work are appropriately investigated and resolved. All persons designated as authors qualify for authorship, and all those who qualify for authorship are listed. Ronan M. G. Berg is the guarantor of this work and accepts full responsibility for the work and the conduct of the study, had access to the data, and controlled the decision to publish.

## CONFLICT OF INTEREST

None declared.

## Data Availability

The data underlying the findings within the present study, can be shared upon reasonable request directed to the corresponding author.

## 1

Tables A1, A2, A3 and Figures A1 and A2.

**TABLE A1:** Quality assessment for reported pulmonary diffusing capacity measurements.

	COPD		Control	
	Upright	Supine	Upright	Supine
Substudy 1				
*n*	33		15	
Total measurements	66	64	30	30
Reported measurements	65	64	30	30
Criterion *A*	59	51	30	28
Criterion *B*	0	0	0	1
Criterion *C*	4	4	0	0
Criterion *D*	0	1	0	1
Criterion *E*	2	8	0	0
Criterion *F*	1	0	0	0
Substudy 2				
*n*	12		12	
Total measurements	24	15	24	24
Reported measurements	24	15	24	23
Criterion *A*	24	14	24	21
Criterion *B*	0	0	0	0
Criterion *C*	0	1	0	0
Criterion *D*	0	0	0	1
Criterion *E*	0	0	0	1
Criterion *F*	0	0	0	0

*Note*: Participants performed the *D*_L,CO,NO_ test in the upright and in the supine postures either across test visits or pre‐ and post a 12‐week training intervention. Each measurement has been evaluated by established guidelines (Nymand et al., 2024). The total measurements represent the total amount of measurements conducted on both visits combined, whereas reported measurements are the number of measurements included in the analysis based on the following criteria.

Criterion A: ≥2 acceptable manoeuvres with fulfilled repeatability → report mean *D*_L,NO_ and *D*_L,CO,5s_ Criterion B: ≥2 acceptable manoeuvres without fulfilled repeatability → report values from the acceptable manoeuvre with highest *D*_L,NO_ Criterion C: 1 acceptable manoeuvre with fulfilled repeatability → report values from the acceptable manoeuvre Criterion D: 1 acceptable manoeuvre without fulfilled repeatability → report values from the acceptable manoeuvre Criterion E: 0 acceptable manoeuvres, but fulfilled repeatability → report mean *D*_L,NO_ and *D*_L,CO,5s_ from repeatable manoeuvres Criterion F: 0 acceptable manoeuvres and no repeatability → failed measurement Abbreviation: COPD, chronic obstructive pulmonary disease.

**TABLE A2:** Internal consistency of diffusing capacity metrics and related measurements between visits 1 and 2 in Substudy 1.

	COPD			Controls		
	Visit 1	Visit 2	*P*	Visit 1	Visit 2	*P*
Upright rest						
*D*_L,NO_ (mmol min^−1^ kPa^−1^)	23 (9)	22 (9)	0.806	37 (8)	35 (8)	0.888
*D*_L,CO,5s_ (mmol min^−1^ kPa^−1^)	5 (2)	5 (2)	0.838	8 (2)	8 (2)	0.784
*D*_M,CO_ (mmol min^−1^ kPa^−1^)	20 (9)	20 (9)	0.922	31 (8)	30 (9)	0.734
*V*_C_ (mL)	39 (16)	37 (17)	0.808	62 (14)	64 (13)	0.796
*V*_A_ (L)	5 (1)	5 (1)	0.915	6 (1)	6 (0)	0.887
BHT (s)	7 (1)	7 (1)	0.562	6 (0)	6 (1)	0.931
$F_{IO_{2}}$ (%)	21 (0)	21 (0)	0.576	21 (0)	20 (0)	0.920
$F_{AO_{2}}$ (%)	17 (1)	17 (1)	0.649	17 (1)	18 (0)	0.956
${\dot{V}}_{O_{2}}$ (mL min^−1^)	754 (252)	741 (252)	0.911	938 (272)	874 (215)	0.770
*V*_I_/VC	98 (7)	96 (7)	0.299	99 (7)	99 (5)	0.856
Supine						
*D*_L,NO_ (mmol min^−1^ kPa^−1^)	21 (9)	21 (10)	0.914	36 (8)	35 (7)	0.720
*D*_L,CO,5s_ (mmol min^−1^ kPa^−1^)	5 (2)	5 (2)	0.837	9 (2)	9 (2)	0.720
*D*_M,CO_ (mmol min^−1^ kPa^−1^)	18 (9)	18 (9)	0.949	28 (7)	27 (8)	0.727
*V*_C_ (mL)	38 (17)	40 (19)	0.717	70 (16)	72 (16)	0.740
*V*_A_ (L)	5 (1)	5 (1)	0.895	6 (1)	6 (1)	0.907
BHT (s)	7 (1)	7 (1)	0.552	6 (1)	6 (0)	0.704
$F_{IO_{2}}$ (%)	21 (0)	21 (0)	0.999	21 (0)	20 (0)	0.562
$F_{AO_{2}}$ (%)	17 (1)	17 (1)	0.735	17 (1)	17 (1)	0.973
${\dot{V}}_{O_{2}}$ (mL min^−1^)	669 (271)	693 (265)	0.898	866 (196)	865 (6)	0.991
*V*_I_/VC	95 (11)	93 (8)	0.583	97 (6)	97 (11)	0.835

*Note*: Absolute values from *D*_L,CO,NO_ measurements in chronic obstructive pulmonary disease (COPD) and matched healthy controls. A linear mixed model with Holm–Bonferroni adjusted *P*‐values was used to test differences between days. Abbreviations: BHT, breath‐hold time; *D*_L,CO,5s_, diffusing capacity for carbon monoxide with 5‐sec breath‐hold; *D*_L,NO_, diffusing capacity for nitric oxide; *D*_M,CO_, alveolar–capillary membrane diffusing capacity for carbon monoxide; $F_{AO_{2}}$, fractional concentration of oxygen in alveolar air; $F_{IO_{2}}$, fractional concentration of oxygen in inspired air; *V*_A_, alveolar volume; *V*_C_, pulmonary capillary blood volume; *V*_I_/VC, ratio between inspiratory volume and vital capacity; ${\dot{V}}_{O_{2}}$, oxygen consumption.

**TABLE A3:** Test–retest reliability indices in Substudy 1.

		**Absolute reliability**			**Relative reliability**					
		**SRD [95% CI] (units)**			**CV [95% CI] (%)**			**ICC [95% CI] (Fraction)**		
**Metric**	**Condition**	**COPD**	**Control**	**Between‐group difference**	**COPD**	**Control**	**Between‐group difference**	**COPD**	**Control**	**Between‐group difference**
*D*_L_ **_, NO_** (mmol min^−1^ kPa^−1^)	Upright	2.9 [2.4, 3.7]	3.9 [2.7, 6.5]	−1.0 [−2.7, 0.9] *P *= 0.140	4.5 [3.4, 5.6]	3.6 [2.3, 4.9]	0.9 [−0.9, 3.0] *P *= 0.151	0.99 [0.98, 0.99]	0.97 [0.87, 0.99]	0.01 [−0.01, 0.05] *P *= 0.114
	Supine	2.7 [2.2, 3.5]	4.2 [3.4, 5.4]	−1.5 [−2.6, −0.3] ***P *= 0.008**	4.5 [3.4; 5.6]	3.9 [2.5, 5.3]	0.6 [−0.9, 2.3] *P *= 0.254	0.99 [0.98, 1]	0.97 [0.89, 0.99]	0.02 [0, 0.07] ***P *= 0.012**
	Upright vs. Supine	−0.2 [−1.1, 0.6] *P *= 0.291	0.3 [−1.6, 2.3] *P *= 0.391		−0.0 [−1.6, 1.7] *P *= 0.486	0.3 [−1.3, 2.3] *P *= 0.360		0 [−0.01, 0.02] *P *= 0.300	−0.01 [−0.03, 0.04] *P *= 0.338	
*D*_L_ **_, CO, 5s_** (mmol min^−1^ kPa^−1^)	Upright	0.8 [0.6, 1.1]	0.9 [0.6, 1.5]	−0.1 [−0.5, 0.4] *P *= 0.413	5.4 [4.1, 6.7]	3.5 [2.3, 4.7]	1.9 [−0.3, 4.4] ***pP *= 0.052**	0.98 [0.97, 0.99]	0.97 [0.91, 0.99]	0.01 [−0.01, 0.06] *P *= 0.234
	Supine	0.8 [0.6, 1.0]	1.3 [1.0, 1.8]	−0.5 [−0.9, −0.1] ***P *= 0.012**	5.4 [4,0, 6.8]	4.9 [3.1, 6.6]	0.5 [−1.4., 2.8] *P *= 0.298	0.99 [0.97, 0.99]	0.94 [0.83, 0.98]	0.1 [0.01, 0.14] ***P *= 0.003**
	Upright vs. Supine	−0.0 [−0.3, 0.2] *P *= 0.428	0.4 [−0.2, 1.0] *P *= 0.087		−0.0 [−2.7, 2.5] *P *= 0.474	1.4 [−0.8, 3.4] *P *= 0.107		0.0 [−0.01, 0.02] *P *= 0.314	−0.03 [−0.1, 0.02] *P *= 0.121	
*D*_M_ **_, CO_** (mmol min^−1^ kPa^−1^)	Upright	3.3 [2.6, 4.4]	6.7 [5.2, 9.0]	−3.4 [−5.3, −1.2] ***P *= 0.000**	5.7 [4.3, 7.1]	7.3 [4.7, 9.8]	−1.6 [−4.5, 1.1] *P *= 0.128	0.98 [0.97, 0.99]	0.94 [0.78, 0.99]	0.05 [0.01, 0.2] ***P *= 0.005**
	Supine	3.6 [2.7, 4.9]	5.2 [4.1, 6.9]	−1.6 [−3.3, −0.1] ***P *= 0.033**	6.9 [5.2, 8.6]	6.2 [4.0, 8.4]	0.7 [−1.7, 3.1] *P *= 0.304	0.98 [0.96, 0.99]	0.95 [0.86, 0.98]	0.03 [0.0, 0.1] ***P *= 0.023**
	Upright vs. Supine	0.2 [−1.2, 1.4] *P *= 0.352	−1.5 [−3.5, 0.8] *P *= 0.094		1.2 [−1.0, 3.2] *P *= 0.125	−1.0 [−4.2, 2.0] *P *= 0.248		0 [−0.02.; 0.02] *P *= 0.365	0.01 [−0.06, 0.12] *P *= 0.344	
*V*_C_ (mL)	Upright	10.1 [8.0, 13.5]	18.8 [15.1, 24.2]	−8.6 [−13.4, −3.46 ***P *= 0.000**	9.1 [6.9, 11.4]	9.8 [6.3, 13.4]	−0.7 [−4.2, 2.8] *P *= 0.333	0.95 [0.91, 0.98]	0.78 [0.46, 0.92]	0.18 [0.07, 0.49] ***P *= 0.001**
	Supine	10.9 [9.4, 13.1]	23.9 [20.5, 28.3]	−13.0 [−17.2, 9.1] ***P *= 0.000**	9.7 [7.3, 12.2]	11.1 [7.1, 15.1]	−1.3 [−4.0, 1.0] *P *= 0.143	0.96 [0.91, 0.98]	0.75 [0.41, 0.91]	0.21 [0.1, 0.6] ***P *= 0.000**
	Upright vs. Supine	0.6 [−2.6, 3.5] *P *= 0.344	5.2 [−0.9, 11.0] ***P *= 0.041**		0.7 [−2.3; 3.7] *P *= 0.347	1.2 [−2.2, 4.6] *P *= 0.232		0.01 [−0.03, 0.06] *P *= 0.352	−0.03 [−0.38, 0.29] *P *= 0.412	
*V*_A_ (L)	Upright	0.43 [0.36, 0.53]	0.27 [0.19, 0.42]	0.16 [0.03, 0.29] ***P* = 0.010**	3.0 [2.3, 3.8]	1.5 [1.0; 2.0]	1.5 [0.7, 2.3] ***P *= 0.000**	0.99 [0.98, 0.99]	0.99 [0.97, 0.99]	0 [−0.02, 0.01] *P *= 0.406
	Supine	0.42 [0.30, 0.64]	0.29 [0.21, 0.44]	0.13 [−0.04, 0.31] *P *= 0.078	3.2 [2.4, 4.0]	1.6 [1.1, 2.2]	1.6 [0.4, 2.8] ***P *= 0.005**	0.99 [0.97, 0.99]	0.99 [0.96, 1]	0 [−0.02, 0.03] *P *= 0.488
	Upright vs. Supine	−0.01 [−0.17, 0.17] *P *= 0.395	0.02 [−0.13, 0.16] *P *= 0.397		0.2 [−1.0, 1.6] *P *= 0.450	0.2 [−0.7, 1.0] *P *= 0.341		0 [−0.02, 0.01] *P *= 0.488	0 [−0.03, 0.01] *P *= 0.327	

*Note*: Participants who completed measurements on both study days are included within the analysis (COPD: Upright, *n* = 33; Supine, *n* = 31; Control: Upright, *n* = 15, Supine, *n* = 15). *P*‐values shown in bold indicate statistical significance (*P* < 0.05). Abbreviations: COPD, chronic obstructive pulmonary disease; CV, coefficient of variation; *D*_L,CO,5s_, diffusing capacity for carbon monoxide with 5‐sec breath‐hold; *D*_L,NO_, diffusing capacity for nitric oxide; *D*_M,CO_, alveolar–capillary membrane diffusing capacity for carbon monoxide; ICC, intraclass correlation coefficient; SRD, smallest real difference; *V*_A_, alveolar volume; *V*_C_, pulmonary capillary blood volume.
